# Informal employment and health status in Central America

**DOI:** 10.1186/s12889-015-2030-9

**Published:** 2015-07-24

**Authors:** María López-Ruiz, Lucía Artazcoz, José Miguel Martínez, Marianela Rojas, Fernando G. Benavides

**Affiliations:** CIBER Epidemiología y Salud Pública (CIBERESP), Madrid, Spain; Center for Research in Occupational Health, Universitat Pompeu Fabra, Barcelona, Spain; IMIM (Hospital del Mar Medical Research Institute), Barcelona, Spain; Facultad Latinoamericana de Ciencias Sociales, Salamanca, Spain; Agència de Salut Pública de Barcelona, Barcelona, Spain; Institute of Biomedical Research (IIB-Sant Pau), Barcelona, Spain; Programa Salud, Trabajo y Ambiente en América Central (SALTRA), Universidad Nacional, Heredia, Costa Rica

**Keywords:** Informal employment, Health inequalities, Central America, Occupational health, Mental health, Self-perceived health, Social security coverage

## Abstract

**Background:**

Informal employment is assumed to be an important but seldom studied social determinant of health, affecting a large number of workers around the world. Although informal employment arrangements constitute a permanent, structural pillar of many labor markets in low- and middle-income countries, studies about its relationship with health status are still scarce. In Central America more than 60 % of non-agricultural workers have informal employment. Therefore, we aimed to assess differences in self-perceived and mental health status of Central Americans with different patterns of informal and formal employment.

**Methods:**

Employment profiles were created by combining employment relations (employees, self-employed, employers), social security coverage (yes/no) and type of contract -only for employees- (written, oral, none), in a cross-sectional study of 8,823 non-agricultural workers based on the I Central American Survey of Working Conditions and Health of 2011. Using logistic regression models, adjusted odds ratios (aOR) by country, age and occupation, of poor self-perceived and mental health were calculated by sex. Different models were first fitted separately for the three dimensions of employment conditions, then for employment profiles as independent variables.

**Results:**

Poor self-perceived health was reported by 34 % of women and 27 % of men, and 30 % of women and 26 % of men reported poor mental health. Lack of social security coverage was associated with poor self-perceived health (women, aOR: 1.38, 95 % CI: 1.13-1.67; men, aOR: 1.36, 95 % CI: 1.13-1.63). Almost all employment profiles with no social security coverage were significantly associated with poor self-perceived and poor mental health in both sexes.

**Conclusions:**

Our results show that informal employment is a significant factor in social health inequalities among Central American workers, which could be diminished by policies aimed at increasing social security coverage.

## Background

Since the term “informal sector” was coined in the early 1970s [1], several, non-mutually exclusive approaches and descriptions of informality have emerged as follows: i) as completely separate from or even the opposite of formal work, where the former provides income for the poor who are excluded from the labor market due to an excess workforce in urban areas [2]; ii) as subordinate and dependent micro enterprises where workers are used by large and formal enterprises to reduce labor costs (mainly taxes and workers’ social coverage) [3]; iii) as a result of micro enterprises that prefer informality over the difficult process of formal regulation in terms of time, costs, and efforts [4]; and iv) as a voluntary option because of the cost benefits of operating in the informality [5].

Concurrently, the informality concept has evolved. Earlier, the term informal sector was defined as “unregistered and/or small-scale private unincorporated enterprises engaged in activities, with at least some of the goods or services produced for sale or barter” [6]. However, the broadest and most completely operational term to refer to informality is that of “informal economy” as adopted by the International Labor Organization (ILO) in 2002. This definition is based on both the economic production units and employment relations, and defined as “all economic activities by workers and economic units that are -in law or in practice- not covered or insufficiently covered by formal arrangements”. Given this definition, all informal employment, inside and outside the informal sector, as well as households, is captured including informal wage employees, employers, self-employed, and members of informal producers’ cooperatives and informal contributing family workers who “are not recognized, registered, regulated, or protected under labor legislation and social protection” [7].

Because of the relative ease of creating new jobs, the informal economy often grows faster during periods of economic crisis [8]. In Central America, the structural adjustment programs adopted during the economic crisis of the 1980s have meant an opening-up of their economies to external markets. This caused the restructuring of the labor market including deregulation of the formal sector, increase of unemployment, and consolidation of the informal economy [7, 9]. All of these changes led to a deterioration of working conditions for most of the working population in Central America [10], especially for informal workers among whom the goal of reaching the criteria for decent work is still far from being achieved [11]. Furthermore, it is important to take into account that women, young people, the elderly, and the poor make up a major portion of the informal economy [7, 12].

The Central American region is noted for significant health inequities arising from working and employment conditions [10]. As in the rest of Latin America, in Central America the informal economy is a permanent, structural pillar of the labor market. In the region, among the different kinds of informal jobs, construction work for men and selling products in the street for women are the most common types [13]. On average, more than 60 % of non-agricultural workers are informally employed, with major differences between countries (70 % in Honduras and around 40 % in Costa Rica and Panamá). Moreover, a higher proportion of women are in informal employment (78 % of women versus 74 % of men in Honduras, 72 % versus 60 % in El Salvador, and 46 % versus 42 % in Costa Rica, respectively) [14].

So far, the study of the informal economy has been focused primarily on its nature, definition, measurement, and on its relationship with decent work. Studies about the relationship between informal employment and health status are still scarce. Most existing studies analyzing this relationship find that workers in the informal economy are more likely to report poor health status [15–20]. However, most previous health research is based on a unidimensional measurement of informal employment; for example, absence of an employment contract, lack of social security, or self-employment [15–21]. These are crucial dimensions of informality according to the measurement recommendations of the ILO [22]. However, health research studies should consider the interactions of these measurements rather than the mere study of each dimension separately or the study of all the dimensions in an aggregated form. Finally, although many previous studies adjusted their analyses for sex [15, 20] thereby assuming that the impact of informal employment on health is similar for men and women, the association between informal employment and health may differ by gender [17], which requires, at the least, analyses stratified by sex [23].

Informal employment is assumed to be an important but seldom studied social determinant of health [24], affecting a large number of workers in Central America. Therefore, our aim was to further the understanding of the relationship between health (self-perceived health and mental health) and informal employment by applying a more sensitive measure of formal and informal employment. In accordance with the ILO recommendations, this new measure combines three potentially interacting dimensions of informal employment; social security coverage, type of contract, and employment relations [22]. Moreover, potential interactions between this new indicator and gender are also explored. The study compares patterns of informal and formal employment in a representative sample of Central American workers.

## Methods

### Sources of information

The data source used in this study was the First Central American Survey of Working Conditions and Health (Spanish acronym: ECCTS - Encuesta Centroamericana de Condiciones de Trabajo y Salud) conducted between July and December 2011. We received permission to use the data from the ECCTS research team, which includes some of the co-authors on this paper [25]. Moreover, the data from the ECCTS are publicly available upon request to the corresponding author, who is also one of the data managers of the dataset. The ECCTS is a cross-sectional study of a representative national sample of 12,024 workers (2,004 per country) performed in Costa Rica, El Salvador, Guatemala, Honduras, Nicaragua, and Panama by completing an interviewer-administered questionnaire in the homes of the participants. The response rate of this survey ranged from around 50 % in Costa Rica, 60 % in Honduras to 80 % in the rest of countries. Benavides et al. give more information about the background of the ECCTS [25].

### Study population

After applying the inclusion criteria, the study population consisted of 8,823 workers engaged in non-agricultural activities, aged 18 years and older, and residing in Central America. Agricultural activities were not included because they must be studied separately due to their own peculiarities and the difficulty in differentiating among informal agricultural work and agriculture of subsistence [22]. Employers with fewer than five employees were included in the study, as a proxy of informal sector [22]. Conversely, employers with five or more employees were not included because of the limited number of cases and, mainly, since it can be assumed that this group finds itself in a distinct employment context requiring it to be studied separately.

### Variables

The main variables were those that characterized the situation of formal and informal employment: employment relations (employees, self-employed, or employers with fewer than five employees), social security coverage (yes or no), and, only for employees, the type of contract (written, oral, or no contract). These three variables were first examined separately. In a second step, in order to explore different situations of formal and informal employment, we defined eight “employment profiles” resulting from the combination of the above-mentioned variables, and following the Hussmanns’ matrix, which provides a conceptual framework for informal employment used by ILO [22].

Data on self-perceived health status were elicited by asking respondents to describe their general health as “very good”, “good”, “fair”, “poor”, or “very poor” [26]. This variable was dichotomized by combining the categories “fair”, “poor”, and “very poor” to indicate poor self-perceived health, and “very good” and “good” to indicate good self-perceived health [27]. Mental health was measured using the 12-Item version of the General Health Questionnaire [28], which is a validated screening instrument that detects psychological distress such as anxiety or depressive symptoms. Mental health was dichotomized into good mental health (a score of less than 4) and poor mental health (a score of 4 or more), following the recommended threshold score for some countries of the Latin America region [29].

The analysis was stratified by sex (women or men), and adjusted for age (categorized into 18 to 30 years, 31 to 50 years, or more than 50 years), country (Costa Rica, El Salvador, Guatemala, Honduras, Nicaragua, or Panama), and occupation (management, scientific technicians, or professionals; support technicians or professionals; clerical workers; services workers; vendors; fisherman, rangers, or farmers; artisans, skilled industrial or machinery operators; or unskilled workers).

### Statistical analysis

Several multivariable logistic regression models were fitted, stratified by sex, in order to calculate odds ratios (OR) with 95 % confidence intervals (CI) for poor self-perceived health and poor mental health. The models were also adjusted for age, country, and occupation (aOR). Firstly, we fit models separately with social security coverage, employment relations, and type of contract for employees as the predictor variables, with the categories of having social security coverage, employees, and written contract as the reference groups, respectively. Finally, models with “employment profiles” as the independent variable were estimated, with the reference group being employees covered by social security and with a written contract as the most formal employment profile. For all models, the number of missing cases did not exceed 2.3 % for self-perceived health, and 7.0 % for mental health status. All the analyses were conducted using the Central America database, and using weights for the Central American region (for age, sex, economic activity, and country) in order to adjust for sample selection [25].

## Results

### Description of the sample

A total of 8,823 Central American workers involved in non-agricultural activities were analyzed (48.5 % women). Less than a quarter of the sample was aged over 50 years. Whereas vendor was the main occupation among almost half of women, artisan, skilled industrial or machinery operator (36.3 %), and vendor (25.9 %) were the main occupations among men (Table 1).

**Table 1:** General description of the sample population. Number (n) and percentage (%) of socio-economic and employment conditions and employment profile variables by sex, in Central America, 2011

	Women		Men		Total	
	n	%	n	%	n	%
**Country**						
Guatemala	1,100	25.7	1,348	29.7	2,448	27.7
El Salvador	681	15.9	748	16.5	1,429	16.2
Honduras	807	18.8	716	15.8	1,523	17.3
Nicaragua	849	19.8	559	12.3	1,408	16.0
Costa Rica	505	11.8	633	13.9	1,138	12.9
Panamá	340	7.9	536	11.8	876	9.9
**Age**						
18-30 years	1,803	42.1	1,768	38.9	3,571	40.5
31-50 years	1,858	43.4	1,991	43.8	3,849	43.6
more than 50 years	620	14.5	783	17.2	1,403	15.9
**Occupation** ^a^						
Management, scientific technicians or professionals	317	7.4	185	4.1	502	5.7
Support technicians or professionals	168	3.9	189	4.2	357	4.0
Clerical workers	421	9.8	264	5.8	685	7.8
Services workers	391	9.1	383	8.4	774	8.8
Vendors	1,985	46.4	1,178	25.9	3,163	25.9
Fishers, rangers or farmers	27	0.6	180	4.0	207	2.3
Artisans, skilled industrial or machinery operators	619	14.5	1,647	36.3	2,266	25.7
Unskilled workers	353	8.2	514	11.3	867	9.8
**Social security coverage** ^a^						
Yes	1,303	30.7	1,493	33.3	2,796	32.0
No	2,937	69.3	2,991	66.7	5,928	68.0
**Employment relations**						
Employees	1,873	43.7	2,166	47.7	4,039	45.8
Self-employed	1,592	37.2	1,416	31.2	3,008	34.1
Employers with fewer than 5 employees	817	19.1	959	21.1	1,776	20.1
**Type of contract (for employees)** ^a^						
Written contract	1,170	62.9	1,284	59.8	2,454	61.3
Oral or without contract	689	37.1	863	40.2	1,552	38.7
**Employment profiles** ^a^						
Employees						
Social security coverage						
Written contract	986	23.3	1,094	24.5	2,080	23.9
Oral or without contract	168	4.0	196	4.4	364	4.2
No social security coverage						
Written contract	183	4.3	189	4.2	372	4.3
Oral or without contract	517	12.2	664	14.9	1,181	13.6
Self-employed						
Social security coverage	89	2.1	116	2.6	205	2.4
No social security coverage	1,468	34.7	1,261	28.2	2,729	31.4
Employers with fewer than 5 employees						
Social security coverage	53	1.3	79	1.8	132	1.5
No social security coverage	761	18.0	867	19.4	1,628	18.7
**Total**	4,282	48.5	4,541	51.5	8,823	100.0

^a^There are 2 missing values for occupation, 99 for social security coverage, 33 for type of contract and 132 for employment profiles

About two thirds of both women and men had no social security coverage. Among employees, 37.1 % of women and 40.2 % of men worked with an oral or no contract. The employment profiles with a higher number of workers were self-employed without social security coverage (34.7 % of women and 28.2 % of men) and employees with social security coverage and a written contract (23.3 % and 24.5 % of women and men, respectively). The next profile was employers with fewer than five employees without social security coverage (18 % of women and 19.4 % of men) (Table 1).

### Prevalence of poor health

The prevalence of poor health outcomes was higher among women. Overall, 33.8 % of the women reported poor self-perceived and 29.7 % poor mental health. In men, 26.8 % reported poor self-perceived health and 26.2 % poor mental health (Table 2). Specifically for employment profiles, the prevalence of poor self-perceived health in women was highest among employers with fewer than five employees and no social security coverage (43 %). This was also the case for self-employed men with no social security coverage (33.6 %). Regarding poor mental health, the highest prevalence in women was found for employees with social security coverage and who had an oral or no contract (35.7 %), and for male employees with no social security coverage and a written contract (32.1 %) (Table 3).

**Table 2:** Number (n), prevalence (%) and associations (odds ratios) of poor self-perceived and poor mental health according to employment conditions by sex in Central America, 2011

	Poor self-perceived health				Poor mental health			
	n	%	OR (95 % CI)	aOR (95 % CI)	n	%	OR (95 % CI)	aOR (95 % CI)
**Women**								
** Social security coverage**								
Yes	331	25.4	1.00	1.00	297	24.5	1.00	1.00
No	1,099	37.5	1.76 (1.52; 2.04)***	1.38 (1.13; 1.67)**	874	31.9	1.44 (1.23; 1.68)***	1.19 (0.98; 1.45)
** Employment relations**								
Employees	495	26.5	1.00	1.00	468	26.9	1.00	1.00
Self-employed	615	38.7	1.75 (1.52; 2.02)***	1.27 (1.05; 1.54)*	502	33.6	1.38 (1.18; 1.60)***	1.09 (0.90; 1.32)
Employers with fewer than 5 employees	338	41.4	1.96 (1.65; 2.33)***	1.33 (1.07; 1.65)*	217	28.4	1.08 (0.89; 1.30)	0.91 (0.72; 1.15)
** Type of contract (for employees)**								
Written contract	287	24.6	1.00	1.00	257	23.4	1.00	1.00
Oral or no contract	203	29.6	1.29 (1.05; 1.60)*	1.47 (1.13; 1.93)**	205	32.7	1.60 (1.29; 1.99)***	1.32 (1.01; 1.71)*
** Total**	1,447	33.8			1,187	29.7		
**Men**								
** Social security coverage**								
Yes	281	18.9	1.00	1.00	306	21.5	1.00	1.00
No	923	31.0	1.94 (1.66; 2.25)***	1.36 (1.13; 1.63)**	799	28.6	1.46 (1.26; 1.70)***	1.15 (0.95; 1.38)
** Employment relations**								
Employees	469	21.7	1.00	1.00	488	24.1	1.00	1.00
Self-employed	452	32.2	1.71 (1.47; 1.99)***	1.22 (1.02; 1.45)*	386	28.9	1.29 (1.10; 1.50)**	1.00 (0.83; 1.20)
Employers with fewer than 5 employees	296	31.0	1.62 (1.37; 1.93)***	1.20 (0.99; 1.45)^a^	244	26.9	1.16 (0.97; 1.39)	1.04 (0.85; 1.27)
** Type of contract (for employees)**								
Written contract	244	19.0	1.00	1.00	257	20.9	1.00	1.00
Oral or no contract	214	24.8	1.41 (1.14; 1.73)***	1.13 (0.88; 1.44)	221	28.2	1.48 (1.20; 1.82)***	1.26 (0.99; 1.61)^b^
** Total**	1,217	26.9			1,117	26.2		

*OR* odds ratio, *95 % CI* 95 % confidence interval, *aOR* adjusted odds ratio for age, country, and occupation

*p-value < 0.05 **p-value <0.01 ***p-value < 0.001

^a^ *p*-value <0.06 ^b^ *p*-value <0.08

**Table 3:** Number (n), prevalence (%) and associations (odds ratios) of poor self-perceived and poor mental health according to non-agricultural employment profiles by sex in Central America, 2011

	Poor self-perceived health				Poor mental health			
	n	%	OR (95 % CI)	aOR (95 % CI)	n	%	OR (95 % CI)	aOR (95 % CI)
**Women**								
Employees								
Social security coverage								
Written contract	250	25.4	1.00	1.00	212	22.9	1.00	1.00
Oral or no contract	37	22.2	0.84 (0.56; 1.24)	1.06 (0.68; 1.63)	51	35.7	1.86 (1.28; 2.71)**	1.68 (1.12; 2.53)*
No social security coverage								
Written contract	37	20.2	0.74 (0.50; 1.09)	0.76 (0.50; 1.14)	44	25.6	1.16 (0.80; 1.69)	1.08 (0.73; 1.60)
Oral or no contract	162	31.5	1.35 (1.07; 1.70)*	1.44 (1.08; 1.91)*	154	32.0	1.59 (1.24; 2.03)***	1.31 (0.99; 1.74)^b^
Self-employed								
Social security coverage	32	36.0	1.62 (1.02; 2.56)*	1.39 (0.82; 2.37)	25	29.4	1.39 (0.85; 2.27)	1.15 (0.67; 1.96)
No social security coverage	571	38.9	1.87 (1.57; 2.23)***	1.53 (1.19; 1.97)***	463	33.7	1.71 (1.42; 2.07)***	1.34 (1.03; 1.73)*
Employers with fewer than 5 employees								
Social security coverage	11	20.8	0.73 (0.37; 1.45)	0.55 (0.26; 1.17)	7	13.2	0.49 (0.22; 1.12)	0.44 (0.19; 1.02)^a^
No social security coverage	327	43.0	2.21 (1.81; 2.71)**	1.70 (1.30; 2.23)***	208	29.3	1.40 (1.12; 1.75)**	1.17 (0.88; 1.56)
Total	1,427	33.8			1,164	29.5		
**Men**								
Employees								
Social security coverage								
Written contract	192	17.6	1.00	1.00	204	19.3	1.00	1.00
Oral or no contract	40	20.4	1.22 (0.83; 1.78)	1.08 (0.72; 1.62)	53	29.6	1.77(1.24; 2.53)**	1.70 (1.16; 2.48)**
No social security coverage								
Written contract	51	27.0	1.76 (1.23; 2.51)**	1.49 (1.03; 2.17)*	54	32.1	1.97 (1.38; 2.82)***	1.90 (1.30; 2.78)**
Oral or no contract	172	25.9	1.64 (1.30; 2.07)***	1.22 (0.94; 1.59)	166	27.7	1.61 (1.27; 2.04)***	1.26 (0.97; 1.65)
Self-employed								
Social security coverage	27	23.3	1.42 (0.90; 2.24)	1.09 (0.66; 1.79)	34	31.8	1.96 (1.27; 3.02)**	1.87 (1.16; 3.01)*
No social security coverage	420	33.6	2.37 (1.95; 2.89)***	1.48 (1.17; 1.87)***	341	28.6	1.68 (1.38; 2.05)***	1.17 (0.92; 1.49)
Employers with fewer than 5 employees								
Social security coverage	19	24.1	1.46 (0.85; 2.51)	1.14 (0.64; 2.03)	13	18.1	0.93 (0.50; 1.72)	0.96 (0.50; 1.86)
No social security coverage	272	31.6	2.17 (1.75; 2.68)***	1.42 (1.11; 1.81)**	231	28.0	1.63 (1.32; 2.03)***	1.31 (1.02; 1.69)*
Total	1,193	26.8			1,096	26.1		

*OR* odds ratio; *95 % CI* 95 % confidence interval, *aOR* adjusted odds ratio for age, country, and occupation

*p-value < 0.05 **p-value < 0.01 ***p-value < 0.001

^a^ *p*-value < 0.06 ^b^ *p*-value < 0.08

### Employment conditions and health

For women, not having social security coverage was significantly associated with poor self-perceived health (aOR: 1.38, 95 % CI: 1.13-1.67). Regarding employment relations and compared with employees, an association with poor self-perceived health was found for self-employed (aOR: 1.27, 95 % CI: 1.05-1.54) and employers with fewer than five employees (aOR: 1.33, 95 % CI: 1.07-1.65). Finally, being employed under an oral or no contract was associated with poor self-perceived health (aOR: 1.47, 95 % CI: 1.13-1.93) and poor mental health (aOR: 1.32, 95 % CI: 1.01-1.71) compared with employees who had a written contract (Table 2).

Men working with no social security coverage were more likely to report poor self-perceived health (aOR: 1.36, 95 % CI: 1.13-1.63). Compared with employees, the only significant associations with poor self-perceived health were found for self-employed (aOR: 1.22, 95 % CI: 1.02-1.45) (Table 2).

### Employment profiles and health

There were almost no gender differences in self-perceived health status among association patterns. For both sexes, and compared to the profile of reference (employees with social security coverage and a written contract), the prevalence of poor self-perceived health status was significantly higher among employees with no social security coverage (with an oral or no contract for women, and with a written contract for men), self-employed with no social security coverage, and for employers with fewer than five employees with no social security coverage (Table 3). Whereas the employment profiles associated with poor mental health among women were employees with social security coverage with an oral or no contract (aOR: 1.68, 95 % CI: 1.12-2.53) and self-employed with no social security coverage (aOR: 1.34, 95 % CI: 1.03-1.73); among men, poor mental health was present in most of the employment profiles, except for employees with no social security coverage with an oral or no contract, self-employed with no social security coverage, and employers with fewer than five employees with social security coverage (Table 3).

## Discussion

This study produced three main findings. The first and most important one is that, when the analysis simultaneously included the three dimensions of informal employment, not having social security coverage was the strongest predictor of poor health status for both women and men. Second, when these dimensions were examined separately, not having social security coverage, being self-employed, and being employed with an oral or no contract, were strongly associated with poor health outcomes in both sexes. Lastly, among employees of both sexes with social security coverage, those with an oral or no contract were more likely to report poor mental health.

Our results are consistent with previous studies, which have found that workers in informal employment settings have poorer health status. One study reported that being an informal worker was associated with common mental disorders compared with formal workers [20]. Other studies also observed that women working in informal employment were more likely to report poor mental health [17, 19]. Another study found that female housemaids (mostly with informal job contracts) had worse mental health indicators, including depression and anxiety symptoms, than women with other occupations (principally those with formal job contracts) [18]. Some studies carried out in South Africa, which constructed a complex formality index [30, 31], have also shown that informal employees were more likely to report poorer health status, although it depends on the interaction with earnings. Finally, another study reported that living in a household with at least one informally employed person was associated with poor self-perceived health status, regardless of individual socioeconomic factors and housing characteristics [15]. However, most prior research on the Latin America region has compared only formal versus informal employment, aggregating several categories of informal employment that can actually have different meanings and therefore different impacts on health [17, 19, 20]. In considering different categories of informal employment, we have identified a lack of social security coverage as a key issue.

There are several non-mutually exclusive mechanisms that might explain the relationship between informal employment and health status. Firstly, it is important to note that our reference employment profile is basically the Fordist notion of standard employment (employees covered by social security with a written contract) [32], which has never represented the main labor market relation paradigm in Central America. Social security coverage and formal employment were more prominent in the region before the economic crisis in the 1980s. Since that time, formalization and salaried processes have declined. The informal economy took hold as a result of unemployment (mainly of formal workers) and the flexibility and precariousness of the labor market, which characterized the neoliberal process of structural adjustment of this crisis coupled with the globalization of markets [33]. In this context, a mechanism that could be operating is the health-related features of the wider phenomenon of employment precariousness. In Central America, there is considerable employment precariousness, particularly affecting informal workers [34].

Employment precariousness is characterized by employment insecurity, economic vulnerability, temporality, low collective bargaining power, low earnings, and a lack of social protections; all of which have the potential to affect a worker’s health [35, 36]. In many cases, these characteristics are present in informal employment, so we cannot rule out the hypothesis that the poor health of informal workers runs through the health-related features of employment precariousness.

Employment precariousness in turn is related to wider social precariousness, including denied access to health care, which may also affect the health status among workers in informal employment. In Central America, between 13 % (Guatemala) and 42 % (El Salvador) of people do not have health service coverage [37]. Informal employment and poverty were among the access barriers identified as well as the structure and organization of health systems, which differentiates countries regarding the quality and the diversity of health services offered and that are accessible to people who need them, as pointed out in different studies [38–40]. Therefore, precarious employment could also result in more precarious lives, and the persistence of poverty and poor living conditions [41, 42].

Secondly, although we adjusted for occupation as a proxy of working conditions, differences in working conditions between formal and informal workers could still persist. Working conditions in informal employment are usually poorer than those in formal employment [43]. This is illustrated by taking the occupation of vendors as an example. In Central America, the percentage of informal vendors far exceeds the percentage of formal ones (75 % versus 25 %, respectively) [34]. Also, in our sample there was a huge proportion of vendors, primarily informal self-employed and informal employers, comprising around 70 % and 72 % of women and 41 % and 34 % of men, respectively. Most of the formal vendors were not working on the street, but almost 20 % of women and 30 % of men who were informal vendors were street vendors *(results not shown).* As Marcelli et al. illustrate [44], “Selling oranges in a grocery store is a formal economic activity. Selling them on a highway exit ramp in Los Angeles County to passing motorists is an informal activity”. Some of the working conditions that may be different between the two kinds of vendors are that street vendors are exposed to long working hours, unsafe workplaces, traffic pollution, musculoskeletal problems, inclement weather, and even sexual harassment among women [45–47]. Lastly, a remarkable difference in working conditions between formal and informal workers, not only in vendors, could be the exposure to long working hours, which have a harmful impact on workers’ health and with different gender patterns [48]. In our study, most of the informal employment profiles were more exposed to long working hours (exceeding 48 h per week) for both, women and men. This is particularly the case of employees with no social security coverage with an oral or no contract (41 % of women and 45 % of men), self-employed with no social security coverage (34 % and 38 % of women and men, respectively), and employers with fewer than five employees with no social security coverage, among whom 48 % of women and 36 % of men worked more than 48 h per week. On the contrary, only 19 % of women and 28 % of men employees with social security coverage and a written contract were exposed to long working hours *(results not shown).* These results are consistent with previous studies which have found a relationship between socioeconomic vulnerability and acceptance of obligatory long working hours and other poor social and economic conditions [49].

Additionally, there is a close relationship between employment and working conditions and living conditions outside of work. Poor working and employment conditions, such as those that often characterize informal employment, are connected to poverty, with occupational hazards and poor living conditions combining in nonadditive ways [50]. Hence, we also suggest that poverty and social exclusion could be another possible mechanism explaining poorer health outcomes among people performing informal work [51–54]. Despite the fact that not all informal workers are poor (one study shows there is a proportion of the informal sector that on average earns more than its formal counterpart [52]), lots of poor workers are informally employed, with an over-representation of women in low- and middle-income countries [12]. Likewise, for a large proportion of people in poverty or on the border of social exclusion, informality is a survival strategy as it is the only way to enter into the labor market. At the start of the century, the informal sector in Latin America accounted for around 70 % of employment among the urban poor (approximately 80 % in Guatemala, Honduras and Nicaragua, 74 % in Panama, 62 % in Costa Rica, and 36 % in El Salvador) [55]. In addition, almost four out of ten households in Central America are estimated to be in a situation of exclusion (approximately three out of ten in urban areas and five out of ten in rural areas). People living in such households enter the labor market through subsistence self-employment (95 %), without social security coverage (more than 99 %), and more than half have not completed primary education [33].

Hence, there could be a vicious circle of informality, labor precariousness, poor working and living conditions and poverty that may generate and perpetuate health inequalities among many Central American workers by the different axes of health inequalities. The employability of certain groups more prevalent in the informal economy, such as women or indigenous people, is even more difficult and is mediated by exclusion dynamics, which makes it very hard for members of these groups to break out of this cycle [56].

Likewise, labor precariousness and poor working conditions do not only affect to informal workers in Central America. The effects of globalization and structural adjustment programs on the quality of employment could also have led to a systemic precariousness of formal employment in the region (specifically for paid employment) and a deterioration of working conditions, regardless of the formal or informal nature of the job [10, 11, 57]. Therefore, they could explain the significantly poorer mental health found for employees with social security coverage and an oral or no contract. Contrarily, there was no significant association with poor self-perceived health in this employment profile. As previously mentioned, informal employment could be a barrier to accessing good quality health services [38–40]. So we suggest that these employees, covered by social security, could more easily access good quality of health services, and therefore they have a better perception of their health. Finally, it is important to notice that this employment profile only accounted for around 4 % of workers, and among them 61 % of women and 53 % of men were from Costa Rica (*results not shown*). Although the analysis were adjusted by country, it may be possible that this finding is showing a specific reality of this country, which should be studied more deeply in the future.

There were small gender differences, either in the prevalence of different types of informality or in the pattern of association between informal employment and poor health status. The prevalence of informal profiles was slightly higher among women, contrary to results from previous research. Future studies will be needed to determine whether the pattern is changing in Central America or whether perhaps the ECCTS is not fully representative of informal employment in the region.

On the other hand, regarding the pattern of association between informality and poor health status, there was only one remarkable gender difference. Female employees, without social security coverage but with a written contract, were not different from the reference category (covered by social security and with a written contract). For men in the same situation, the odds of poor health outcomes were significantly higher. Possible explanations for this finding could be the interplay among the axes of social inequality in provoking health inequalities. In this case, gender inequalities could be mediated by social class, as these women are clearly in a more favorable social position than men in the same employment profile. Whereas a large proportion of these women were in non-manual or skilled occupations (56.6 %), men were more often represented in manual and unskilled ones (47.2 %). In addition, 60.2 % of the women had a university or secondary education versus only 41 % of the men. Moreover, the number of weekly working hours also differed. While 21.7 % of women worked more than 48 h per week, 35.4 % of men worked more than 48 h. Finally, another noteworthy result is that 61.8 % of women were young, aged between 18 and 30 years old, unlike the men with only 44.8 % were in this age group *(results not shown).*

It is well-known that there are large health inequalities by gender, but they also depend on interactions with social class and other axes of inequality [58, 59]. In our study, the employment profile of employees without social security coverage but with a written contract applied to groups whose characteristics differed by gender, in that it was more favorable for women, who had better health than men (probably due to their more favorable social class, as noted above). Therefore, the meaning of these dimensions, as well as their influence on health, differed by gender and social class. This employment profile is an interesting example, as the observed differences may be due to these women being in a more favorable social class than the men with this employment profile, as most of them are young women with high education and skilled non-manual jobs, who have good health.

In order to fully understand the gender dimension of informality and its impact on health, future studies should examine the interaction between informal employment and family characteristics, since their relation with the labor market for women, often through informal employment, is still mediated by the woman’s role at home and the general socioeconomic situation of the household [41]. For example, one study shows that 21.7 % of women in Latin America with the highest household incomes only engage in unpaid work (at home) in contrast with 46.5 % of women in lower income households [60]. This study also reported that in Honduras, the labor market participation rate of women from households with no children under six years old is 42.6 %, while for those having three or more children in that age range, the rate decreased to 26.6 % (differences were seen regarding educational level, as the participation rate of the latter women is 23.3 % for those with less than four years of education, but 72.6 % for those with thirteen or more years of education). Finally, the interaction and the intersectionality between informal employment, gender and other axes of inequalities such as age group, immigration [12], ethnicity [61], social exclusion [62], and territory [63] should also be analyzed in detail in future studies [64].

Of note, social class is also a mechanism that could be operating not only on the gender differences observed, but transversally in the different mechanisms involved. Since formal employment in Central America remains exceptional, the selection into formal employment is mainly subject to a medium and high level of social closure. In other words, it is practically reserved for individuals of an advantaged social class, well-positioned in society, and enjoying resources that facilitate their life course, including access to higher education, health care, and good living conditions [65]. As a consequence, these workers enjoy a better health status. Therefore it is possible that this category of formal workers, as the reference employment profile, is in fact a highly selective well-off sample. This could partly explain the strong health inequalities found. In that sense, the distinction between formality and informality could become a proxy for class inequalities. Just as informality is the path for many of the poor, formality could be the path for many of the favored social classes.

Furthermore, a main characteristic of this reference employment profile of formal workers is the availability of social security coverage, with the security and all the job benefits that it represents for them including retirement pension, paid vacations, sick leave, maternity/paternity leave, weekends off, personal/family leave, or breastfeeding time for women. The ILO strategy of formalization of employment highlights the importance of extending social protections to all workers to reach the goal of decent work in the immediate term [7]. Promotion of this strategy for the working poor has also shown that it constitutes a key pathway to reduce poverty and improve their working and living conditions, with an emphasis on women because of their large numbers in the informal economy [12]. Therefore, if we consider that work is a central element of life for many people, giving them access to economic resources and opportunities for achieving good health [66], it would be essential to encourage decent work by the extension of social security coverage for all workers.

### Strengths and limitations

Among the strengths of the study, it is important to remark that, as far as we know, this is the first time that reliable and homogeneous information has been gathered in Central America about informal employment and health status. Most research has neglected the potential association with poor health status. Moreover, the study is based on a large and representative sample of Central American workers. Additionally, we have constructed a complex employment profile with different dimensions of formal and informal employment, which advances our understanding of the complex universe of informality instead of simply dichotomizing between formal and informal employment. Nevertheless, this study has several limitations. For example, we could not exactly apply the classification proposed by ILO [22] since the survey did not allow us to distinguish between contributing family workers or members of producers’ cooperatives for jobs, or between households for the type of production unit. Moreover, we had to use a proxy of the informal sector (fewer than five workers) because the questionnaire did not ask about the legal registry of the company. Furthermore, we could not separate employees according to different informal sectors because of the limited sample size. Since this is a cross-sectional study, we cannot rule out the possibility of reverse causation, whereby rather than the experience of informal employment leading to poor health status, it may be that people with poorer health are more likely to work in informal job arrangements. Moreover, people with good health may be more likely to work in formal employment due to their favorable social status. Finally, since the analysis was carried out for the entire Central American region, we cannot rule out potential differences between countries derived from their political and cultural differences. Despite this, our results are consistent with our hypotheses, and we may assume that they could be transferable across countries of the region. Nevertheless, future studies will have to be performed but using countries separately in order to deepen the specificities of each one.

## Conclusions

Addressing informality is essential because informal employment arrangements constitute a permanent, structural of the labor market in Central America and other low- and middle-incomes countries. These informal workers are not protected by the prevailing employment legislation. This study is a first approach to informal employment as a determinant of workers’ health in Central America including gender as a variable potentially involved in interactions. It reinforces most previous research reporting that workers in informal employment have a poorer health status than those formally employed. According to our results, the lack of social security coverage is probably the most important dimension linking informal employment with poor health in the region. Accordingly, universal social protection is conceived as a priority not only for improving working conditions or to eradicate poverty, but for improving workers’ health and reducing social inequalities in health, neutralizing the most harmful consequences of informality, trying to formalize their situation, and improving their living conditions.
